# 15-year ultra-late presentation of bifocal hepatic metastasis from gastrointestinal stromal tumor: a case report

**DOI:** 10.3389/fsurg.2026.1788993

**Published:** 2026-04-01

**Authors:** Yong Hou, Shijie Zhong, Hongan Chen, Fan Cao, Anshu Xu

**Affiliations:** 1 Minimally Invasive Hepatobiliary Surgery, Qujing Central Hospital of Yunnan Province, Affiliated Qujing Hospital of Kunming Medical University, Qujing, Yunnan Province, China; 2 Yiling Hospital of Yichang, Affiliated Yiling Hospital of China Three Gorges University, Yichang, Hubei Province, China

**Keywords:** gastrointestinal stromal tumor (GIST), hepatic metastasis, imatinib, recurrence, surgical treatment

## Abstract

Gastrointestinal stromal tumors (GISTs) are rare mesenchymal neoplasms of the gastrointestinal tract, and ultra-late recurrence (defined as recurrence occurring ≥10 years postoperatively) is particularly uncommon, especially among patients with inadequate adjuvant targeted therapy. Herein, we report a 64-year-old male patient with jejunal GIST who developed bifocal hepatic metastases 15 years after primary tumor resection. Postoperatively, the patient received only 1 year of adjuvant imatinib (400 mg/day) and remained disease-free for the subsequent 14 years, until liver lesions were incidentally identified during a routine physical examination. Comprehensive imaging studies confirmed two lesions in liver segments S4 (43 mm) and S7 (17 mm), which were consistent with metastatic characteristics. The patient subsequently underwent partial hepatectomy of segments S4 and S7 with intraoperative ultrasound-guided wire localization, achieving R0 resection. Pathological examination of both metastatic lesions revealed spindle cell proliferation, with immunohistochemical staining positive for CD117 (+) and Dog-1 (+), consistent with the typical phenotypic profile of GIST, thus confirming their GIST origin. At the 6-month postoperative follow-up, the patient remained asymptomatic, with no radiological or clinical evidence of tumor recurrence. This case is clinically distinctive owing to its ultra-long recurrence interval and synchronous bifocal hepatic metastasis, thereby offering valuable insights into the long-term natural course of GISTs in the setting of inadequate adjuvant tyrosine kinase inhibitor (TKI) therapy. Furthermore, it underscores the necessity of long-term postoperative surveillance for patients with GIST and highlights the potential role of comprehensive genetic testing in guiding individualized treatment decisions.

## Introduction

Gastrointestinal stromal tumors (GISTs) are the most common mesenchymal tumors of the gastrointestinal tract. Owing to their non-specific clinical manifestations, GISTs are often frequently incidentally detected on imaging or endoscopic examinations (1). For localized GISTs, surgical resection is the main treatment method, while for high-risk or metastatic cases, tyrosine kinase inhibitors (TKIs) are the standard adjuvant treatment (2, 3). The most common sites of GISTs metastasis are the liver and peritoneum (4). Studies have shown that GISTs metastasis usually occurs in the early stage after diagnosis, especially when the tumor has high proliferative activity or adverse pathological features (5). Ultra-late recurrence (defined as metastasis occurring more than 10 years after surgery) is extremely rare (6), but due to its extremely low incidence, relevant clinical data are still scarce. Especially for patients with inadequate adjuvant targeted therapy, there is a lack of clear data on their long-term natural course.

This article reports a rare case of a patient with jejunal GIST who developed bifocal liver metastases 15 years after surgical resection of the primary lesion. The patient received only one year of adjuvant imatinib therapy postoperatively and remained disease-free for the subsequent 14 years. The hepatic lesions were eventually discovered during a routine physical examination. The uniqueness of this case lies not only in its ultra-late recurrence interval and bifocal hepatic metastasis, but more importantly, in that it provides valuable data regarding the long-term natural history of GISTs in the setting of inadequate adjuvant TKIs therapy.

## Patient information

The patient is a 64-year-old male who was admitted to our hospital with a 15-year history of small intestinal stromal tumor resection and a liver mass identified one day prior to admission. The patient and his family stated that he undergone surgery for a small intestinal stromal tumor 15 years ago and recently a space-occupying lesion in the liver was found during a regular physical examination. On admission, the patient was asymptomatic and denied symptoms including cough, expectoration, abdominal pain, abdominal distension, dizziness, headache, palpitation, chest tightness, shortness of breath, nausea, vomiting, hematemesis, melena, frequent urination, urgent urination, dysuria, chills and fever. The patient had a good mental status, with normal diet and sleep, regular bowel and bladder movements, and no significant weight change recently.

## Past medical history and medication history

The patient had a 2-year history of coronary heart disease and had been taking oral aspirin once daily, one tablet per administration. He also had an 8-year history of hypertension and had been receiving oral amlodipine besylate tablets combined with rosuvastatin calcium tablets, with his blood pressure being well-controlled. The patient denied a personal history of diabetes, cerebrovascular disease, viral hepatitis, tuberculosis, typhoid fever, malaria, mental disorders and other comorbidities, and had no history of traumatic injury. In 2010, the patient underwent surgical resection for a jejunal stromal tumor at the Department of General Surgery, our hospital. After the operation, he took imatinib orally once a day, 400 mg each time, and stopped taking the drug one year later. The patient denied a history of blood transfusion and drug allergy, and his vaccination history was unobtainable.

## Review of the disease course and timeline

In 2010, the patient was diagnosed with “small intestinal stromal tumor” after undergoing abdominal enhanced CT in our hospital. Subsequently, under general anesthesia, the patient underwent “intestinal resection and anastomosis”. During the operation, a 10 × 8 × 6 cm mass was found 30 cm from the Treitz ligament in the jejunum, which was closely adhered to the intestinal tube and mesentery. No other significant postoperative complications occurred, and the patient was discharged following an uneventful recovery. The pathological biopsy of the resected tissue is as follows. In 2025, the patient was readmitted to our hospital after a routine physical examination revealed a hepatic space-occupying lesion.

The results of the puncture biopsy are as follows:

1. (Small intestine) Considered to be gastrointestinal stromal tumor (see paraffin)
2. No tumor invasion was found at both ends of the resection.

## Physical examination on admission in 2025

On admission, the patient's vital signs were as follows: pulse, 83 beats per minute; respiratory rate, 20 breaths per minute; blood pressure, 136/75 mmHg. On abdominal physical examination, a 10-cm curvilinear old surgical scar was identified on the abdomen. The abdomen was soft and flat, with no tenderness or rebound tenderness on palpation. Hepatomegaly and splenomegaly were absent on palpation. The shifting dullness was negative. No percussion tenderness was noted in the hepatic or costovertebral angles.

## Laboratory and imaging examination results

**Figure 1:**
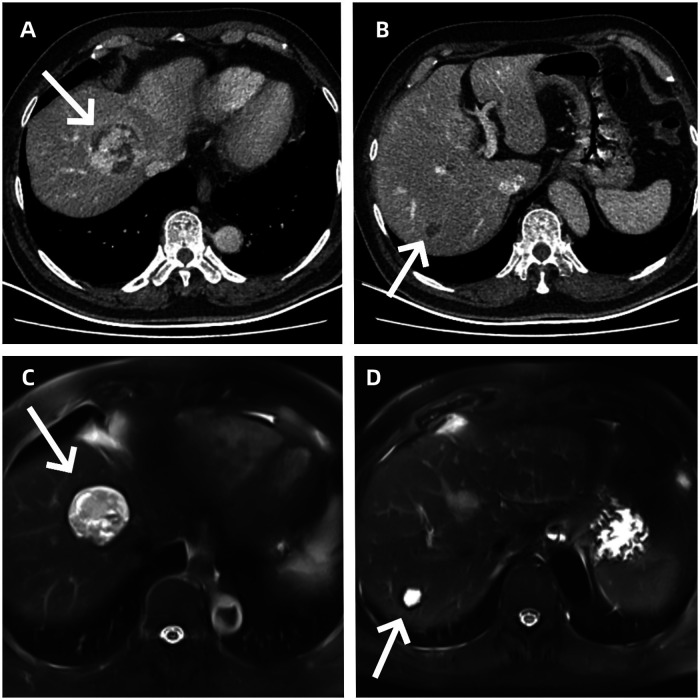
**(A)** enhanced CT shows a tumor in the left medial lobe of the liver. **(B)** Enhanced CT shows a tumor in the lower segment of the right posterior lobe of the liver. **(C)** MRI shows a tumor in the left medial lobe of the liver. **(D)** MRI shows a tumor in the lower segment of the right posterior lobe of the liver.

Following admission to the hospital, the patient completed a comprehensive set of imaging examinations and laboratory tests. Portal vein computed tomographic venography (CTV) combined with contrast-enhanced whole-abdominal CT revealed slightly hypodense masses in hepatic segments S4 (45 mm) and S7 (15 mm), which exhibited a typical enhancement pattern of metastatic lesions after contrast administration (Figures 1A,B); whole-abdominal magnetic resonance imaging (MRI) further confirmed the presence of soft tissue masses in hepatic segments S4 (43 mm) and S7 (17 mm), consistent with the features of metastatic lesions (Figures 1C,D).

## Diagnostic assessment

Preoperative diagnosis: malignant hepatic neoplasm (suspected stromal tumor recurrence), stage 2 hypertension, gastric polyps, colon polyps, chronic gastritis, gallbladder polyps, status post jejunal stromal tumor resection, benign prostatic hyperplasia, hepatic cysts, and pulmonary opacities. Based on the patient's clinical history, the diagnosis was confirmed as ultra-late hepatic metastasis from gastrointestinal stromal tumor. Surgical intervention was therefore recommended for the patient.

## Treatment

**Figure 2:**
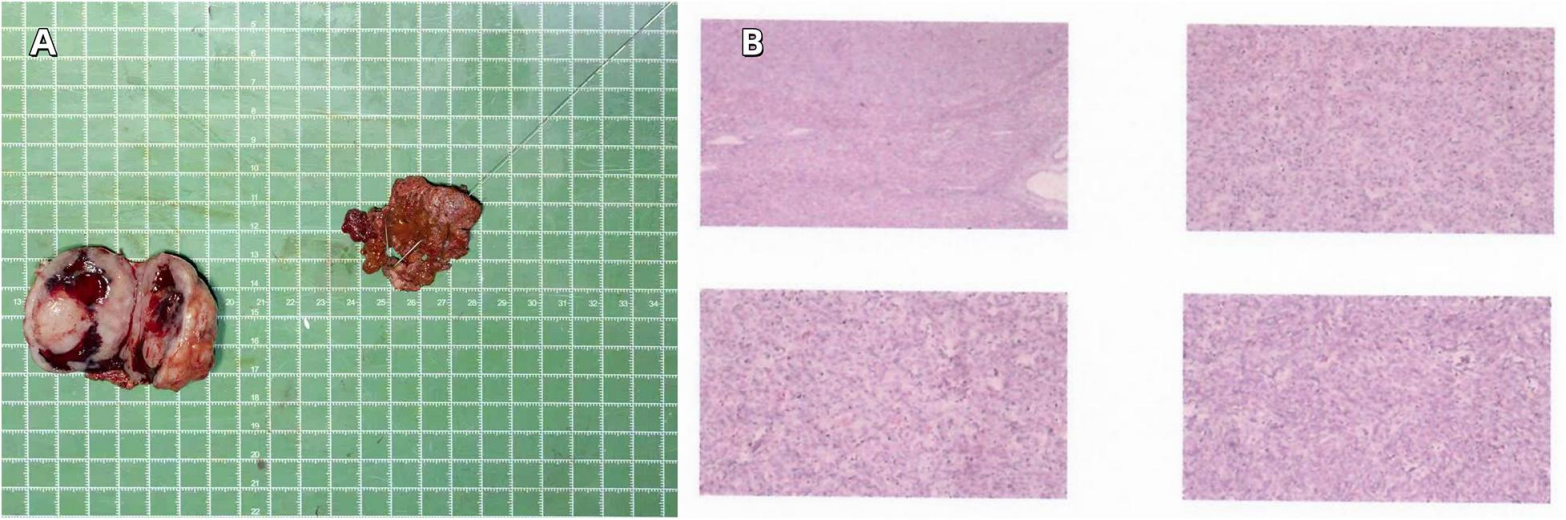
**(A)** resected liver tumor. **(B)** Pathological section of liver metastasis from gastrointestinal stromal tumor.

After detailed informed consent with the patient and his family, a partial hepatectomy of hepatic segments S4 and S7 was performed under general anesthesia on May 30, 2025. During the operation, a 5.0  × 5.0 × 4.0 cm solid mass was identified in hepatic segment S4, whereas no obvious lesions were palpable in hepatic segment S7. Subsequently, intraoperative ultrasound (IOUS) identified a 1.9 × 1.5 × 1.4 cm lesion in hepatic segment S7, which was precisely localized with a guidewire under real-time guidance and completely resected (see Figure 2B). Postoperative pathological examination confirmed the following findings: hepatic mass (S4): gastrointestinal stromal tumor, consistent with metastatic disease in the context of the clinical history. Hepatic mass (S7): gastrointestinal stromal tumor, consistent with metastatic disease in the context of the clinical history. Immunohistochemical staining results were as follows: PCK (-), Vimentin (+), Ki-67 (15%+), CD117 (+), DOG-1 (+), CD34 (focal +), CD31 (vascular +), P53 (+), F-Ⅷ (vascular +), Bcl-2 (+), CD99 (focal +), HMB-45 (-), MelanA (-), S-100 (-), Desmin (focal +), SMA (-).

Postoperative blood routine examination and related biochemical index tests were conducted on the patient. The results showed mild hyperglycemia and a decrease in hemoglobin levels, while no significant abnormalities were found in hepatic and renal function. Concurrently, the patient underwent genetic testing. The assay was performed using next-generation sequencing (NGS) on the Illumina sequencing platform (NextSeq500/NovaSeq6000). Genetic testing results identified the following variants: 1. A variant with strong clinical significance (Class I): A p.A502_Y503 duplication mutation in exon 9 of the KIT gene, which is an activating mutation; 2. A variant with potential clinical significance (Class II): A heterozygous mutation in the RB1 gene. In the early postoperative period, the patient underwent chest and abdominal CT scans, which revealed pleural effusion and atelectasis. No other postoperative complications occurred, and the patient was discharged successfully on June 10, 2025. Subsequent to discharge, the patient was initiated on oral imatinib therapy at a daily dose of 400 mg, with a recommendation for lifelong administration.

## Follow-up and outcomes

The patient has been followed up to December 2025. During this period, multiple imaging evaluations were conducted.

On June 17, 2025, plain CT scan of the upper abdomen revealed patchy hypodense areas in the surgical field following partial hepatectomy. The volume of intra-abdominal and pelvic fluid collections, as well as intra-abdominal free gas, was reduced compared with prior imaging studies; bilateral pleural effusions and pulmonary infiltrates showed mild improvement.

On July 8, 2025, ultrasound demonstrated coarsened and increased echogenicity of the residual liver parenchyma and right-sided pleural effusion.

On October 16, 2025, Contrast-enhanced CT of the entire abdomen demonstrated a slight increase in encapsulated fluid collections and pneumoperitoneum at the surgical site compared with the prior scan, whereas intra-abdominal and pelvic fluid collections had completely resolved. No definitive evidence of tumor recurrence was identified.

On December 4, 2025, follow-up ultrasound confirmed persistent coarse echotexture of the residual liver parenchyma and a 22 mm × 21 mm hyperechoic lesion in the right posterior hepatic lobe, consistent with postoperative changes.

At the time of the most recent follow-up, the patient remained in stable general condition without symptoms such as abdominal pain or distension. Liver and renal function tests, as well as tumor marker levels, remained within stable limits, with no clinical or radiological indications of local recurrence or distant metastasis.

## Discussion

Compared with previously reported cases of ultra-late metastasis, the uniqueness of this case lies in the dual rarity of both the timing and pattern of recurrence. The patient had undergone surgical resection for primary jejunal GIST followed by only one year of adjuvant imatinib therapy, with hepatic metastasis emerging 14 years after treatment discontinuation. This prolonged interval far exceeds the typical recurrence window for GIST, in which the majority of recurrences occur within the first five years post-resection (6). This case parallels the report by Grossi et al. (7) of a gastric GIST presenting liver metastasis 23 years after surgery. Notably, the metastatic lesions in this instance exhibited a synchronous bilobar distribution, in contrast to most documented cases of ultra-late metastasis, which typically present as solitary hepatic lesions, synchronous multifocal involvement is exceedingly rare. This pattern expands the clinical phenotypic spectrum of recurrence in patients with very advanced GISTs.

In clinical practice, the standard duration of adjuvant imatinib therapy for intermediate-risk and high-risk GIST patients is usually 3 years (8), and the ESMO guidelines more explicitly recommend 400 mg/d of adjuvant imatinib for 36 months in high-risk patients (9). However, in this case, the patient only completed 1 year of adjuvant therapy and then stopped the medication, yet achieved 14 years of disease-free survival. This phenomenon merely reflects the unique indolent biological heterogeneity potentially exhibited by a subset of GISTs and does not invalidate the duration of adjuvant therapy for high-risk GISTs as recommended in current clinical guidelines. Management of such cases requires a comprehensive assessment incorporating genotypic profiling and tumor pathological characteristics, and this clinical scenario cannot be generalized for routine clinical practice. For the majority of patients with high-risk GISTs, long-term adjuvant imatinib therapy remains the standard regimen for reducing recurrence risk. This case represents a rare clinical scenario and lacks sufficient evidentiary support to alter the current clinical treatment paradigm for GIST. Additionally, the patient was asymptomatic at presentation, and the hepatic lesions were identified only during a routine physical examination, which further underscores the importance of regular imaging surveillance during long-term follow-up for GIST patients. Current clinical guidelines recommend that high-risk GIST patients undergo imaging assessments every 3 to 6 months for the first 3 years postoperatively, followed by annual imaging surveillance thereafter (9).

Both hepatic metastases at S4 and S7 presented as spindle cell proliferation, with immunohistochemical staining showing CD117 (+) and DOG-1 (+), which is consistent with the typical phenotype of GIST. Approximately 95% of GISTs can detect KIT (CD117) expression, and CD117 and DOG-1 are reliable diagnostic markers for GIST (10). Furthermore, the pathological features of the metastases were consistent with those of the primary lesion, confirming the homology of the metastases. In this case, the Ki-67 proliferation index of the metastases was 15%, indicating that the tumor cells had a certain proliferative activity but were in a long-term dormant state, which is inconsistent with the traditional understanding that “a high proliferation index indicates a poor prognosis” (11). Genetic testing revealed that the patient simultaneously carried a KIT gene exon 9 duplication mutation (Class I strong clinical significance variant) and an RB1 gene exon 25 inactivating mutation (Class II potential clinical significance variant). The occurrence of GIST is mainly driven by activating mutations in the KIT or PDGFRA genes, with KIT mutations occurring in about 80% of cases and PDGFRA mutations in about 5% (12). The incidence of KIT exon 9 mutations in GIST is only 10% to 13% (13), and in previous ultra-late metastatic cases, KIT exon 11 deletion mutations were predominant - KIT exon 11 mutations are usually associated with a higher risk of recurrence (14). In the absence of paired genetic testing between the primary and metastatic lesions, it remains unclear whether the RB1 mutation was a germline mutation or an acquired somatic mutation following tumor recurrence. The potential synergistic interaction between the RB1 and KIT mutations requires further validation with additional clinical and experimental data. The co-occurrence of RB1 tumor suppressor gene mutations and KIT driver mutation provides a novel clinical case that may inform and advance research into the molecular subtyping of GISTs. This genetic profile differs from the predominant “single driver mutation” pattern reported in previous GIST molecular studies (13, 15), thus providing a valuable reference for investigating the molecular mechanisms underlying long-term tumor dormancy and metastatic activation in GISTs.

In terms of treatment, with the improvement of the safety of hepatectomy, the surgical value of GIST liver metastases (LMs) has been widely recognized. For patients with metastatic GIST to the liver, surgery should be adopted whenever possible (16). Conventional radiotherapy and chemotherapy have poor therapeutic effects on GIST (17). Xiao, B found in a retrospective study specifically targeting patients with liver metastases from gastrointestinal stromal tumors that R0 resection followed by continuous imatinib significantly prolonged the survival of GLM patients, regardless of the extent of the disease or the stage of metastasis (18). Sutton (19) et al. found in a single-center retrospective study that liver resection aimed at cure was associated with improved overall survival in patients with GIST liver metastases. Recently, Wen (20) et al. compared the local treatment value of GIST liver metastases and different surgical methods. They found that the benefits of hepatectomy were significantly better than those of imatinib alone, and achieving a disease-free state through liver surgery could significantly prolong overall survival, liver-specific progression-free survival (PFS). In this case, the surgical approach of “intraoperative ultrasound-guided wire localization + staged hepatic segmentectomy” was adopted, successfully achieving R0 resection of the two metastatic foci, and the patient was disease-free in the short term after surgery. However, there is currently controversy over the duration of imatinib use after surgery. Most studies recommend lifelong medication because it can only inhibit but not eradicate residual microscopic lesions, and short-term medication cannot achieve long-term control. Postoperative occult metastases are common in patients with liver metastases, and discontinuation of treatment can lead to rapid disease progression, while lifelong medication can continuously inhibit lesion proliferation and maximize the extension of progression-free survival (21).

This case also provides new insights into the risk assessment system for GIST recurrence. Contrary to the previous understanding that “KIT exon 9 mutation is equivalent to a worse prognosis” (22), this case implies that this mutant subtype may exhibit the unique biological behavior of “long-term dormancy”. However, in the absence of direct molecular biological evidence (e.g., detection of dormancy-associated gene expression), this interpretation remains speculative. Additionally, other potential contributing factors—such as the slow proliferation of micrometastatic lesions—cannot be excluded. Clinically, the recurrence risk should be comprehensively judged by combining the co-mutation status, treatment history, etc. For similar patients carrying KIT exon 9 mutation + tumor suppressor gene co-mutation, even if they have long-term disease-free survival after surgery, they should be alert to the possibility of ultra-late recurrence to avoid insufficient follow-up due to an initial underestimation of the risk.

This study, as a single-case report, has certain limitations: Firstly,it is not possible to precisely determine the actual activation time of micrometastatic lesions. The so-called “ultra-late recurrence” may have been detected with a delay due to interruptions in surveillance. Secondly, only short-term follow-up data have been obtained so far. The long-term prognosis of the patient and whether there will be a second recurrence still need to be further tracked. Long-term follow-up is of crucial importance. In the future, a multi-center database for ultra-late recurrence of GIST can be established, which can be carried out to provide more sufficient evidence for formulating individualized drug withdrawal and follow-up plans.

## Data Availability

The original contributions presented in the study are included in the article/Supplementary Material, further inquiries can be directed to the corresponding author.
